# Post-Translational Modification of p62: Roles and Regulations in Autophagy

**DOI:** 10.3390/cells14131016

**Published:** 2025-07-02

**Authors:** Shuai Xiao, Yeping Yu, Meng Liao, Dandan Song, Xiaozhen Xu, Lingli Tian, Rui Zhang, Hao Lyu, Dong Guo, Qi Zhang, Xing-Zhen Chen, Cefan Zhou, Jingfeng Tang

**Affiliations:** 1 National “111” Center for Cellular Regulation and Molecular Pharmaceutics, Hubei University of Technology, Wuhan 430068, China; xiaoshuai825@hbut.edu.cn (S.X.); 102210687@hbut.edu.cn (Y.Y.); 102400600@hbut.edu.cn (M.L.); 102300789@hbut.edu.cn (D.S.); 102300803@hbut.edu.cn (X.X.); 102200631@hbut.edu.cn (L.T.); zhangrui1987@hbut.edu.cn (R.Z.); haolyu@hbut.edu.cn (H.L.); jk1103@whu.edu.cn (D.G.); zhangqi@hbut.edu.cn (Q.Z.); 2 Hubei Key Laboratory of Industrial Microbiology, Hubei University of Technology, Wuhan 430068, China; 3 Membrane Protein Disease Research Group, Department of Physiology, Faculty of Medicine and Dentistry, University of Alberta, Edmonton, AB T6G 2R3, Canada; xzchen@ualberta.ca

**Keywords:** post-translational modifications, autophagy, p62, oligomerization

## Abstract

Autophagy is a highly conserved cellular process that plays a crucial role in maintaining cellular homeostasis by degrading damaged organelles, misfolded proteins, and other cellular components. p62/SQSTM1 functions as a selective autophagy receptor by binding polyubiquitinated cargo through its UBA domain and linking it to microtubule-associated protein light chain 3 (LC3)-decorated autophagosomes. Moreover, p62 acts as a signaling hub and is essential in response to various stressors, including nutrient deprivation and oxidative stress. Post-translational modifications (PTMs) critically regulate p62’s multifaceted roles, controlling p62’s phase separation, cargo recruitment, signaling interactions, and autophagic degradation efficiency. The dysregulation of p62 PTMs is closely related to the occurrence and development of human diseases, particularly neurodegenerative disorders and certain cancers. This review summarizes the main PTM events of p62 discovered to date that influence the autophagy process, including phosphorylation, acetylation, ubiquitination, and S-acylation, as well as their known contributions to protein aggregation and disease. The PTMs of p62 dynamically regulate autophagy, protein aggregation, and cellular signaling, underscoring its importance as a potential therapeutic target and biomarker for these diseases.

## 1. Introduction

**Figure 1:**
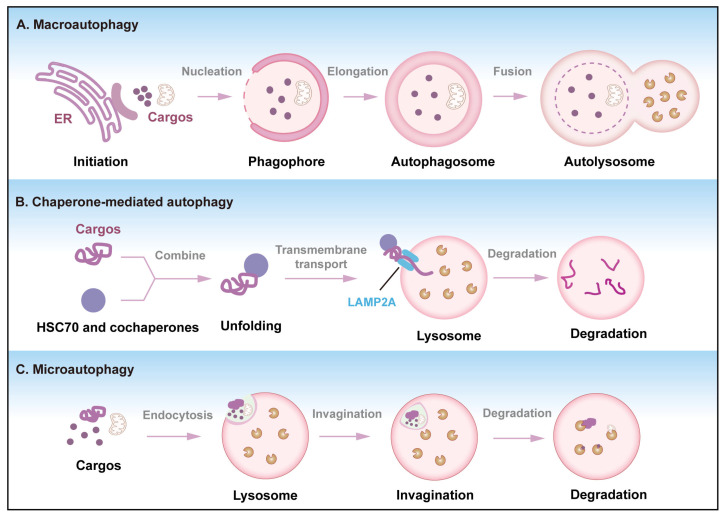
The three different types of autophagy. (**A**) Macroautophagy: Macroautophagy is the most common form of autophagy within cells, which can be divided into four stages: formation of the phagophore, formation of the autophagosome, transport and fusion of the autophagosome, and degradation of the autophagosome. (**B**) Chaperone-mediated autophagy: Substrate proteins directly enter the lysosome for degradation with the assistance of molecular chaperones such as LAMP2A. (**C**) Microautophagy: The lysosomal membrane undergoes deformation and directly takes the substrates in the cytoplasm into the lysosome for degradation. The invagination of the lysosome directly engulfs the cytoplasmic components.

Autophagy is an evolutionarily conserved lysosomal degradation pathway that responds to nutrient deficiencies, energy deficits, protein misfolding, and organelle damage [1]. Autophagy can be categorized into three main types: macroautophagy, microautophagy, and chaperone-mediated autophagy (Figure 1) [2]. Macroautophagy, often referred to as autophagy, is characterized by the encapsulation of degradation products within a double-membrane structure known as autophagosomes, which subsequently fuse with lysosomes to form autolysosomes where degradation occurs [3]. Microautophagy involves the direct encapsulation and degradation of small molecules in the cytoplasm through the invagination of the lysosomal membrane, which can be activated by signal molecules on the surface of damaged organelles [4]. Chaperone-mediated autophagy is a selective process whereby molecular chaperones recognize specific intracellular target proteins and transport them to lysosomes for degradation [5]. While autophagy was initially regarded as a stochastic process, accumulating evidence establishes its selectivity as a fundamental feature. This selective autophagy requires precise substrate recognition mediated by specialized selective autophagy receptors, such as p62/SQSTM1, FUNDC1, NBR1, and TAX1BP1 (Table 1) [6,7,8].

The multifunctional scaffold protein p62 is a classical selective autophagy receptor, which plays a pivotal role in protein quality control [43]. As a selective autophagy receptor, p62 can bind to selected cargo and LC3 [44]. The dual binding functionality ensures efficient cargo sequestration within double-membraned autophagosomes and subsequent delivery to autolysosomes for degradation by acidic hydrolases. The level of p62 inversely correlates with autophagic flux, establishing its utility as a dynamic biomarker for monitoring autophagic degradation [45]. Furthermore, p62 involves the regulation of multiple signaling pathways, including the selective autophagy pathway, NF-κB signaling pathway, Nrf2–Keap1 signaling pathway, mTOR signaling pathway, and others [46,47,48]. Deregulation of p62 is often associated with a variety of diseases, especially cancer, neurodegenerative diseases, and inflammatory diseases [49,50,51]. These findings collectively establish p62 as a master regulator of cellular homeostasis in selective autophagy and signal integration.

Post-translational modifications (PTMs) encompass covalent biochemical alterations after protein biosynthesis, including amino acid side chain modifications and terminal group processing. These enzymatic modifications (including phosphorylation, acetylation, ubiquitination, SUMOylation, and others) dynamically regulate protein stability, subcellular localization, macromolecular interactions, and functional states through specific chemical group additions or removals [52]. p62’s dual roles as an autophagy receptor and signaling hub are tightly regulated through distinct PTMs, including phosphorylation, acetylation, ubiquitination, and S-acylation [53,54,55]. For example, phosphorylation enhances p62 binding to ubiquitinated substrate binding, acetylation modulates p62 oligomerization states, and S-acylation controls p62 liquid–liquid phase separation dynamics. These modifications collectively regulate p62’s capacity to bridge ubiquitinated cargoes with autophagosomal membranes, nucleate self-polymerizing condensates through PB1 domain interactions, and coordinate crosstalk between autophagy and major signaling pathways. In this review, we systematically summarize the ways in which the multifunctionality of p62 is critically regulated by key PTMs, including phosphorylation, ubiquitination, acetylation, and S-acetylation. Specifically, we clarify the mechanistic effects of these PTMs on p62’s functional roles in selective autophagy, diverse signaling pathways, and cellular stress responses. Finally, we explore the dynamic regulation of p62 oligomerization by PTMs, elucidating their dual impact on autophagy modulation and stress adaptation mechanisms.

## 2. Overview of Autophagy

Autophagy is a highly conserved cellular degradation pathway characterized by dynamic membrane reorganization, which progresses through several distinct stages: initiation of phagophore formation, nucleation and elongation of the isolation membrane, maturation into a double-membrane autophagosome, and fusion with lysosomes for cargo degradation [56]. The initiation of autophagy is triggered by various stimuli, including nutrient deprivation, hypoxia, and cellular stress [57,58]. ULK1 complex promotes the formation of the phagophore, which originates from the endoplasmic reticulum (ER), mitochondria, and the recycling endosome, etc. [59]. Then, the expansion of the phagophore involves the elongation and closure of the membrane, ultimately forming a double-membrane autophagosome that encapsulates the targeted cytoplasmic components [60]. The mature autophagosome subsequently fuses with lysosomes to form an autolysosome [61]. Mature autophagosomes dock with lysosomes through SNARE complexes in bulk autophagy, while selective autophagy may employ distinct fusion machinery [62,63]. Once the autophagosome fuses with the lysosome, the encapsulated materials undergo lysosomal degradation and are released back into the cytoplasm for reuse. This recycling process is vital for maintaining cellular energy levels and supporting biosynthetic pathways, particularly under conditions of nutrient scarcity.

**Figure 2:**
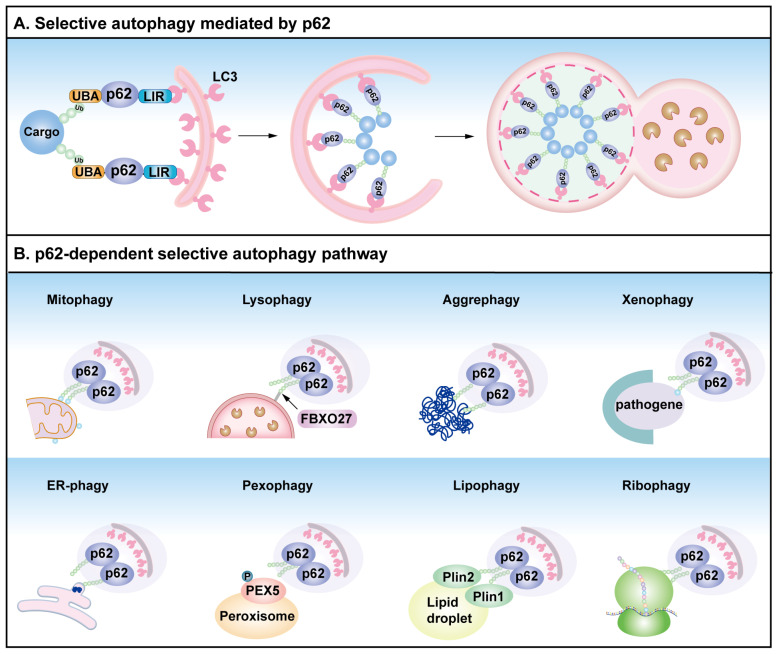
The autophagy receptor p62 and p62-mediated selective autophagy. (**A**) Selective autophagy mediated by p62. As a selective autophagy receptor, p62 links ubiquitinated cargo proteins by the UBA domain, while the LC3-interacting region (LIR) motif interacts with ATG8 family proteins to form autophagic membranes. Subsequently formed autophagosomes fuse with lysosomes to degrade the substrate. (**B**) p62 participates in multiple types of autophagy, including mitophagy, lysophagy, aggrephagy, xenophagy, endoplasmic reticulum (ER)-phagy, pexophagy, lipophagy, and ribophagy.

Mechanistically, autophagy is tightly regulated by a hierarchical network of autophagy-related (ATG) proteins and signaling pathways [59]. In mammals, core ATG proteins orchestrate autophagosome formation through sequential recruitment to pre-autophagosomal structures [64], including the UNC-51-like kinase 1 (ULK1) initiation complex (ULK1–FIP200–ATG13–ATG101), the PI3K complex I, ATG12–ATG5 conjugation systems, and LC3 lipidation machinery [65,66,67]. The AMPK–mTOR–ULK1 axis is a central autophagy induction regulatory hub [68,69]. Under metabolic stress, AMP-activated protein kinase (AMPK) activation phosphorylates ULK1 (Ser317/Ser777) to initiate autophagy [70], while nutrient sufficiency activates mTOR to suppress ULK1 via phosphorylation at Ser757 [71]. This dual regulation ensures precise adaptation to cellular energy status. According to substrate specificity, autophagy can be classified into two types: selective and non-selective. Non-selective autophagy randomly engulfs cytosol during starvation to replenish nutrients [72]. Conversely, selective autophagy employs receptors (p62, NDP52, OPTN) that simultaneously bind ubiquitinated cargo (damaged organelles, protein aggregates) and LC3 on phagophores [73]. p62 is a crucial selective autophagy receptor that facilitates the elimination of damaged organelles and protein aggregates. It plays an essential mediator in several types of selective autophagy, including mitophagy (elimination of mitochondria), pexophagy (peroxisome removal), ER-phagy (the clearance of ER), ribophagy (the clearance of ribosome), lysophagy (damaged lysosome clearance), and nucleophagy (the clearance of nuclear component) (Figure 2) [73,74]. The p62-mediated degradation mechanism is essential for maintaining cellular homeostasis, highlighting the significance of p62 in selective autophagy.

## 3. Structure and Function of p62

**Figure 3:**
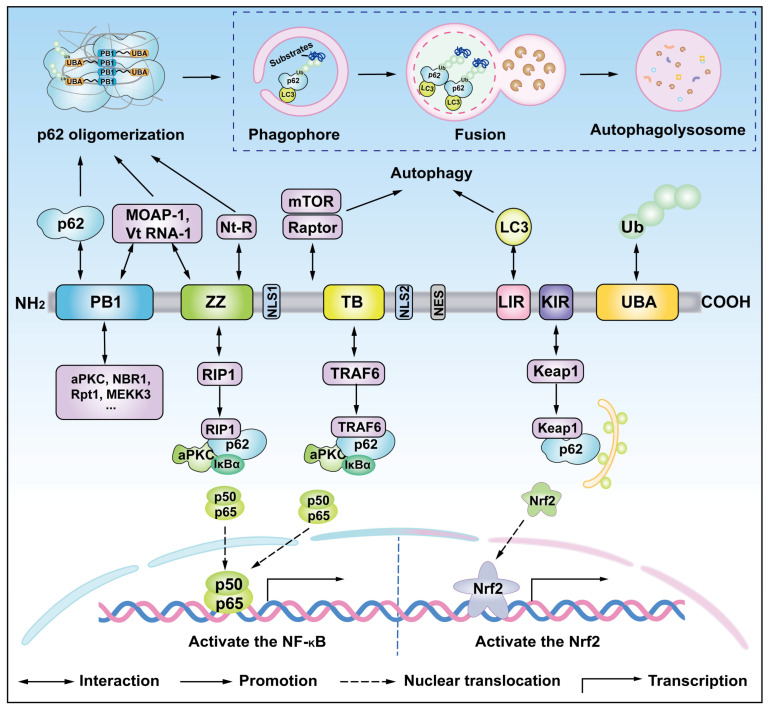
The functional domains of the p62 protein. This figure illustrates the different domains of p62 and the proteins interacting with them, as well as the important functions of these domains. The PB1 domain at the N-terminal of p62 interacts with proteins containing the PB1 domain to promote the formation of homologous or heterologous aggregates. Moreover, the PB1 domain interacts with αPKC, MEKK3, ERK1, and NBR1, thereby controlling important physiological processes, such as the NF-kB pathway, mTORC1 activation, and adipogenesis. The ZZ domain, an atypical zinc finger, binds to RIP kinase to activate the NF-κB pathway. The TB domain interacts with the E3 ubiquitin ligase TRAF6 and thereby regulates the NF-κB pathway. The region between the ZZ and TB domains contains the binding site for the subunit Raptor of the mTORC1 complex, the nuclear localization signals (NLS1/2) and the nuclear export signals (NESs) mediate the nuclear-cytoplasmic shuttling of p62, and the autophagy marker LC3II recognizes and binds to p62 through the LIR domain to promote the formation of autophagosomes. KIR, the Keap-interacting region, binds to the E3 ubiquitin ligase Keap1, thereby activating Nrf2 to cope with oxidative stress. The C-terminal UBA domain is an important region for p62 to bind to ubiquitinated proteins for autophagic degradation. P62 mediates the autophagic degradation of ubiquitinated proteins through the LIR and UBA domains.

p62 is a 440 amino acid protein and contains multiple domains, including the N-terminal Phox and Bam1p domain (PB1 domain), the ZZ-type zinc finger domain (ZZ domain), the tumor necrosis factor receptor-associated factor 6 (TRAF6 domain) binding domain (TB domain), the LC3 interaction domain (LIR domain), the Keap1 interaction domain (KIR domain), and the C-terminal ubiquitin (Ub)-related domain (UBA domain) (Figure 3) [75]. In addition, p62 also has two nuclear localization signals (NLSs) and a nuclear export signal (NES). The multiple structural domains of p62 underlie its diverse functional roles [76].

The PB1 domain drives self-oligomerization through complementary electrostatic interactions between the acidic surface residue Asp69 and basic residue K7 in adjacent PB1 domains [77,78]. This oligomerization capacity allows p62 to form filamentous structures (p62 bodies) that sequester ubiquitinated cargo for autophagic degradation. The PB1 domain further mediates heterotypic interactions with PB1-containing signaling proteins, including mitogen-activated αPKCζ, MEKK3, and NBR1, enabling p62 to regulate NF-κB activation, apoptosis, and selective autophagy [48,79,80]. Adjacent to the PB1 domain, the ZZ domain interacts with RIP1 to connect TNFα signaling to NF-κB activation through TRADD/RIP/p62/aPKC/IKKβ complexes [81]. This domain also recognizes N-terminal arginine residues (N-degrons) via its zinc finger architecture, coupling the Arg–N-degron pathway to autophagic clearance [82]. The PB1 and ZZ domains also regulate the interaction between p62 and LC3 by modulating the exposure of LIR sequences [83]. The central TB domain facilitates Ras-induced carcinogenesis by recruiting TRAF6 to activate mTORC1 and NF-κB pathways, while the juxtaposed Raptor-binding region links p62 to mTORC1-mediated autophagy suppression [84]. The region between the ZZ and TB domains serves as the interaction site for the mTORC1 complex subunit Raptor, which activates the mTORC1 complex and inhibits autophagy activity [85].

The LIR and UBA domains coordinate cargo recognition and autophagosome engagement. The UBA domain selectively binds K63-linked polyubiquitin chains on protein aggregates and organelles [86,87,88], while the LIR motif directly recruits LC3-family proteins to anchor ubiquitinated substrates to autophagic membranes [9]. This dual recognition system ensures efficient cargo sequestration and degradation. Notably, PB1-mediated oligomerization enhances UBA domain avidity for polyubiquitin, creating phase-separated condensates that optimize autophagic flux [89]. The KIR domain engages the Kelch β-propeller of Keap1, competitively displacing Nrf2 to activate antioxidant response elements under oxidative stress [90]. This interaction is regulated by nucleocytoplasmic shuttling via two nuclear localization signals (NLS1/2 and NES), enabling p62 to modulate both cytoplasmic autophagy and nuclear proteostasis [51]. Phosphorylation events at NLS motifs enhance nuclear import, dynamically adjusting p62’s subcellular distribution [76]. Through these coordinated domain interactions, p62 integrates stress signaling (NF-κB, Nrf2), mTORC1, and protein quality control systems. Its structural plasticity allows context-dependent recruitment of kinases (MEKK3, PKCζ), ubiquitin ligases (TRAF6), and chaperones to maintain cellular homeostasis, with dysregulation contributing to cancer, neurodegeneration, and metabolic disorders (Figure 3).

## 4. Post-Translational Modifications of p62

### 4.1. Phosphorylation of p62

**Figure 4:**
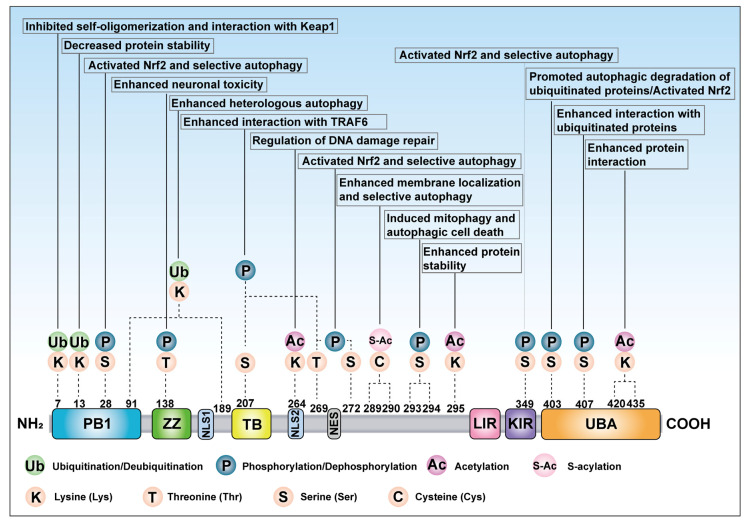
The overview of PTM sites of p62 and the regulated biological functions. There are various PTMs on the p62 protein, such as phosphorylation, dephosphorylation, ubiquitination, deubiquitination, acetylation, and S-acylation. K indicates Lysine at different sites, T indicates Threonine at different sites, S indicates Serine at different sites, and C indicates Cysteine at different sites. The PTMs at different sites of p62 regulate the corresponding biological functions.

**Figure 5:**
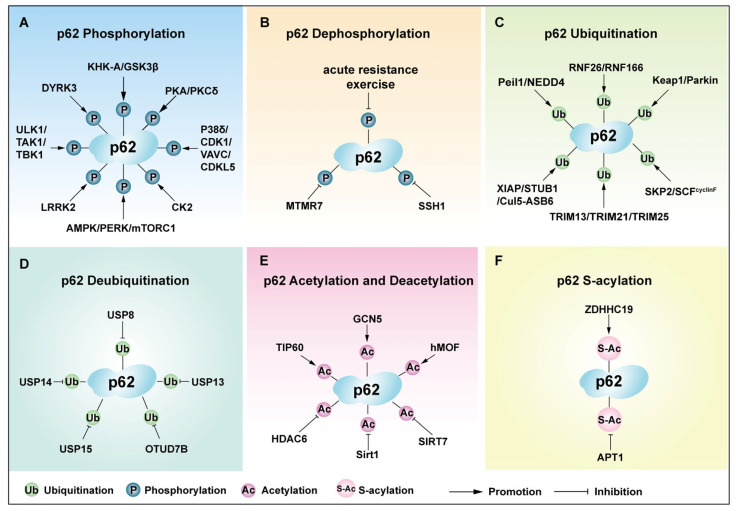
Regulators are involved in the post-translational modification and regulation of p62. (**A**) Regulatory factors affecting the phosphorylation of p62. (**B**) Regulatory factors affecting the dephosphorylation of p62. (**C**) Regulatory factors affecting the ubiquitination of p62. (**D**) Regulatory factors affecting the deubiquitination of p62. (**E**) Regulatory factors affecting the acetylation/deacetylation of p62. (**F**) Regulatory factors affecting the S-acetylation of p62.

Phosphorylation is a key PTM that regulates cellular processes, particularly in autophagy [91]. Phosphorylation of p62 occurs at multiple serine and threonine residues, dynamically altering its structure, interactions, and functional roles (Figure 4 and Figure 5). Specific residues identified include Ser28, Ser293, Ser349, Ser403, Thr269, and others (Table 2).

#### 4.1.1. p62 Phosphorylation by ULK1

ULK1 is identified as a key kinase responsible for the phosphorylation of p62, playing a critical role in autophagy regulation and cellular stress responses [95]. It directly interacts with and phosphorylates p62 at Ser351 (Ser349 in human), promoting the formation of p62 bodies [95]. This process retains Keap1 and activates Nrf2, thereby regulating antioxidant responses [95]. Under proteotoxic stress, ULK1 phosphorylates p62 at Ser405 (in mouse) or Ser403 (in human), along with Ser409 (Ser407 in human), within its UBA domain. This phosphorylation event significantly enhances p62’s affinity for ubiquitinated proteins and polyQ-Htt, promoting their selective clearance via autophagy [103,107]. Furthermore, Sestrin2 facilitates ULK1-mediated phosphorylation of p62 at Ser403, thereby reinforcing the autophagic degradation of p62-bound substrates, including ubiquitinated proteins and protein aggregates [103]. This mechanism underscores p62’s critical role as an autophagy receptor for targeted substrate degradation. Collectively, ULK1-mediated phosphorylation of p62 represents a critical regulatory mechanism that enhances autophagic processes under stress conditions, with therapeutic potential for targeting autophagy-related disorders.

#### 4.1.2. p62 Phosphorylation by TBK1

TANK-binding kinase 1 (TBK1) is a serine/threonine kinase and regulates various biological processes [137]. The phosphorylation of p62 by TBK1 is a critical regulatory mechanism primarily impacting autophagy, mitophagy, and innate immune responses. TBK1 phosphorylates p62 specifically at Ser405 (Ser403 in human) and Ser409 (Ser407 in human) within its UBA domain [108,138]. TBK1 is recruited to p62-containing protein aggregates and directly phosphorylates p62-Ser403, which is crucial for the efficient engulfment and clearance of these aggregates via selective autophagy/mitophagy [102]. Loss-of-function mutations in TBK1 impair p62-Ser403 phosphorylation and consequently disrupt its ubiquitin-binding function [102]. Phosphorylation of p62 significantly enhances its affinity for ubiquitinated cargoes, including proteins tagged for degradation and damaged mitochondria [139]. TBK1 inhibitors, such as BX795, MRT67307, or amlexanox, significantly reduce p62-Ser403 phosphorylation [138]. Dysregulation of TBK1-mediated p62 phosphorylation is linked to neurodegenerative diseases like Amyotrophic Lateral Sclerosis (ALS) and Frontotemporal Lobar Degeneration (FTLD) [140]. Restoring mitophagy through mechanisms activating the TBK1–p62 phosphorylation axis demonstrates neuroprotective potential [140]. When saturated fatty acids induce lipotoxic injury, TBK1-mediated p62 phosphorylation induces ubiquitinated protein aggregation and large protein inclusions, which ultimately promote the development of nonalcoholic steatohepatitis and liver cancer [141]. Palmitic acid (PA) recruits TBK1 to induce p62 Ser403 phosphorylation and aggregation, which activates the non-canonical p62–Keap1–Nrf2 antioxidant pathway [78]. Tiliroside inhibits TBK1 enzymatic activity to decrease the phosphorylation of p62 Ser349 and p62-mediated Keap1 degradation, thereby promoting Keap1-mediated Nrf2 ubiquitination and degradation [96]. Moreover, the TBK1–p62 axis intersects with innate immune signaling. TBK1 phosphorylates p62 to promote the autophagic degradation of ubiquitinated STING, thereby negatively regulating the cGAS–STING pathway upon activation [142].

#### 4.1.3. P62 Phosphorylation by TAK1

Transforming growth factor-β-activated kinase 1 (TAK1) is a serine/threonine protein kinase that functions as a signaling hub in innate immunity and pro-inflammatory signaling pathways [143]. The phosphorylation of p62 by TAK1 is a key mechanism to regulate autophagy, anti-oxidative stress, and inflammatory signals. Research indicates that TAK1 directly influences the phosphorylation of p62 at the Ser351 site (Ser349 in human), facilitating p62-mediated Keap1 selective autophagic degradation and activating Nrf2 nuclear translocation and increasing the expression of multiple antioxidant proteins in the mouse model [144,145]. Moreover, TAK1 enhances p62 phosphorylation at Ser403 enhances the interaction between the UBA domain and polyubiquitin chains in response to stress conditions [104]. It also reduces p62 localization to autophagosomes, inhibiting p62-mediated autophagy substrates [104]. Therefore, TAK1 serves as a pivotal kinase that regulates p62 phosphorylation, which in turn modulates autophagy and antioxidant responses through its interactions with Keap1 and ubiquitinated substrates.

#### 4.1.4. P62 Phosphorylation by PKCδ

Protein kinase C delta (PKCδ) is a member of the PKC family of serine/threonine kinases, which are involved in regulating diverse cellular processes such as apoptosis, differentiation, and signal transduction [146]. PKCδ is directly implicated in the phosphorylation or promotion of p62 phosphorylation. Specifically, PKCδ acts alongside other kinases like CK1 and TAK1 in pathways associated with muscle physiology and stress responses [144]. Phosphorylation of p62 mediated by PKCδ enhances Nrf2 activation, which prevents Nrf2 degradation and promotes its antioxidant activity [147,148]. VPS34 recruits PKCδ to phosphorylate p62 at Ser349, stabilizing Nrf2 to support tumor growth in breast cancer [99]. Furthermore, Licochalcone A inhibits arthritis by activating the p62-Keap1–Nrf2 axis through Ser349 phosphorylation, enhancing antioxidant signaling to alleviate inflammatory damage [149]. Collectively, the phosphorylation of p62 by PKCδ primarily enhances its role as a signaling hub in the Nrf2 antioxidant pathway. This modification activates Nrf2 nuclear translocation and upregulates antioxidant proteins, contributing to cellular defense against oxidative stress.

#### 4.1.5. P62 Phosphorylation by Other Kinases

Under oxidative stress, phosphorylated p62 serves as a critical regulator of the Keap1–Nrf2 pathway by facilitating the sequestration of Keap1 within p62 bodies [150]. This releases Nrf2, enabling its nuclear translocation and subsequent transcription of cytoprotective genes [150]. Multiple kinases regulate p62 phosphorylation at specific sites to modulate this axis. For instance, PKA phosphorylates p62 at Ser349, directly activating the Nrf2 antioxidant response [97]. PERK phosphorylates p62 at Ser351 (Ser349 in human) to activate the non-canonical Keap1–Nrf2 pathway, providing defense against ER stress and ROS [100]. Furthermore, the anti-rheumatic compound Sinomenine induces phosphorylation of p62 at Ser351(Ser349 in human) and Thr269/Ser272 sites in rheumatoid arthritis synovial fibroblasts, which activates Keap1-Nrf2 signaling to enhance its anti-arthritic effects [151]. Oxidative stimulation induces phosphorylation of p62 at Ser28 by KHK-A in hepatocellular carcinoma cells (HCCs), thereby enhancing p62’s aggregation with Keap1 and Nrf2 activation [93]. Moreover, leucine-rich repeat kinase 2 (LRRK2) interacts with p62 and influences its phosphorylation state at Ser351(Ser349 in human) and Ser403, consequently inhibiting the interaction of p62 with Keap1 [101]. Therefore, site-specific phosphorylation of p62 is critical for antioxidant and inflammatory responses.

Phosphorylated p62 critically regulates its function in cargo recognition, autophagosome formation, and integration with key signaling pathways. For instance, casein kinase 2 (CK2) phosphorylates p62 at Ser403, enhancing its ability to bind and concentrate polyubiquitinated substrates [106]. This phosphorylation promotes p62 phase separation, facilitating the formation of liquid droplets that recruit autophagy machinery components like LC3 and concentrate ubiquitinated cargo for efficient autophagic degradation [106]. Concurrently, AMPK phosphorylates p62 at Ser293/Ser294, driving its translocation to mitochondria and activating mitophagy to eliminate dysfunctional organelles, which induces mitophagy and autophagic cell death [94]. The E3 ubiquitin ligase Smurf1 further amplifies p62’s phase separation capability by promoting p62 phosphorylation at Ser349 via mTORC1 activation, which enhances competitive binding to Keap1 and subsequent Nrf2 activation, thereby linking p62-mediated autophagy to antioxidant responses in an mTORC1-dependent manner [98]. The MEKK3–p38δ kinase axis phosphorylates p62 to recruit the ubiquitin ligase TRAF6, which is essential for mTORC1 activation in response to amino acid signaling [152]. Moreover, p38δ phosphorylates p62 at Thr267/Ser272 sites and promotes p62-mediated selective autophagy process under proteasomal stress [111]. Thus, site-specific phosphorylation dynamically tunes p62’s receptor activity, phase separation capacity, and crosstalk with nutrient-sensing pathways to orchestrate selective autophagy.

p62 phosphorylation at specific residues critically influences disease mechanisms and therapeutic strategies. DYRK3-mediated phosphorylation of p62 at Thr269/Ser207 enhances its interaction with TRAF6, activating mTORC1 signaling to drive melanoma progression by promoting tumor growth and metastasis [110]. Cell cycle-dependent kinase 1 (CDK1) phosphorylates p62 at Thr269/Ser272, enhancing cell cycle progression and tumorigenesis in response to Ras-induced transformation [112]. In neurodegenerative contexts, LRRK2 exacerbates neuronal toxicity by phosphorylating the Thr138 site of p62 [109]. Furthermore, LRRK2 amplifies neuroinflammation and ferroptosis in Parkinson’s disease, partly through modulation of the p62–Keap1–Nrf2 pathway [101]. During viral infections, phosphorylation can also regulate p62’s antiviral functions. Phosphorylation at Thr269/Ser272 (by VACV kinase during poxvirus infection) triggers p62 nuclear translocation, evading autophagic degradation to facilitate viral replication [113]. Conversely, in HCMV infection, CDKL5-mediated p62 phosphorylation at Ser272 promotes nuclear retention, restricting viral progeny release by enhancing interactions with viral replication machinery [114,153]. In addition, GSK3β phosphorylates p62 at Ser28, which is critical for p62 degradation under poly (I:C)-induced conditions [92]. Collectively, these modifications dictate p62’s roles in autophagy, stress defense, signaling, and viral restriction, with significant implications for cancer, neurodegenerative diseases, infections, and inflammatory disorders. Future research should focus on kinase-specific inhibitors and in vivo models to exploit p62 phosphorylation therapeutically.

### 4.2. Dephosphorylation of p62

Dephosphorylation of p62 is a critical regulatory step in cellular signaling, autophagy, and stress response pathways. This process is mediated by specific phosphatases. For instance, slingshot protein phosphatase 1 (SSH1) catalyzes the dephosphorylation of p62 at the Ser-403 residue through its C-terminal domain, which inhibits autophagic flux by deactivating p62 as an autophagy receptor and potentially affecting cargo degradation, including the clearance of tau aggregates [116]. Additionally, myotubularin-related protein 7 (MTMR7) induces p62 dephosphorylation, which facilitates its release from mTORC1 [115]. This dissociation prevents p62-mediated glycolytic and phenotypic changes in VSMCs, highlighting p62 dephosphorylation as a regulatory mechanism for cellular proliferation and differentiation [115]. In neurodegenerative disorders (ALS and FTLD), the mutation p62^G427R^ abolishes phosphorylation of Ser351(Ser349 in human) and disrupts p62-mediated selective autophagy, which contributes to impaired clearance of misfolded protein aggregates and accelerates disease progression [108]. Furthermore, acute resistance exercise results in decreased phosphorylation of p62 at Ser403, underscoring the dynamic nature of p62 regulation in response to physiological stimuli and cellular stress responses [154]. Overall, the dephosphorylation of p62 serves as a pivotal switch in cellular homeostasis, elucidating p62 dephosphorylation mechanisms may reveal novel therapeutic targets for restoring proteostasis in neurodegeneration and other stress-related pathologies.

### 4.3. Ubiquitination of p62

Ubiquitination is a critical PTM that is pivotal in regulating various cellular processes, including protein degradation, signal transduction, and cellular homeostasis [155]. Numerous E3 ubiquitin ligases dynamically regulate ubiquitination of p62 through site-specific modifications, influencing its functions in selective autophagy, signaling pathways, and stress responses. The ubiquitination of p62 is critical for its function as an autophagy receptor, as it binds ubiquitinated cargo and directs it to autophagosomes for degradation. For instance, RING finger protein 26 (RNF26) ubiquitinates p62 to recruit vesicle adaptors for cargo transport [125]. RNF166 ubiquitinates p62 at K91/K189 to enhance oligomerization and ligand binding, thereby promoting xenophagy and the removal of pathogens [120]. Conversely, tripartite motif containing-25 (TRIM25) interacts with p62 and promotes its K63-linked ubiquitination during porcine reproductive and respiratory syndrome virus (PRRSV) infection, impairing p62 oligomerization and autophagy and inhibiting protein aggregate degradation [123]. Moreover, ubiquitination promotes the proteasomal degradation of p62. For instance, Parkin ubiquitinates p62 at K13, thereby enhancing proteasomal degradation of p62 [119]. Dysregulation of the parkin–p62 axis may be associated with Parkinson’s disease [119]. Additionally, ubiquitylation of p62 regulates cellular inclusion body autophagy. For instance, NEDD4 and Keap1 drive p62 ubiquitination to promote p62-mediated inclusion body autophagy [117,122]. E3 ligase complex SCF^cyclinF^ ubiquitylates p62 at K281, promoting the aggregation of p62 into the insoluble, whereas mutant cyclin F promotes aberrant p62 ubiquitination and insolubility in ALS and frontotemporal dementia (FTD) Pathogenesis [121].

Furthermore, p62 ubiquitination also influences other cellular signaling pathways, like Keap1/Nrf2 and mTORC1 signaling. For instance, TRIM13 mediates p62 ubiquitination via the Keap1–Nrf2 pathway, triggering autophagy in lung adenocarcinoma cells [126]. MiR-1246 reduces p62 ubiquitination by SKP2, stabilizing p62 and activating Nrf2 signaling, thus promoting the transcription of antioxidant enzymes and relieving reproductive senescence [124]. TRIM21 directly interacts with and catalyzes p62 K63-linked ubiquitination at K7, inhibiting p62 oligomerization and sequestration of Keap1, thus negatively regulating the antioxidant response [118]. STUB1 promotes K48-linked ubiquitination and proteasomal degradation of p62, which activates mTORC1 signaling and T follicular helper cell differentiation in rheumatoid arthritis [127]. Additionally, p62 ubiquitination is associated with human disease. For instance, SERPINH1 inhibits TRIM21-mediated K63-linked ubiquitination of p62, thereby stabilizing p62 and promoting prostate cancer proliferation [156]. The X-linked inhibitor of apoptosis protein (XIAP)-mediated ubiquitination of p62 suppresses autophagy, promoting breast/colorectal cancers [128]. The Cul5/ASB6 complex mediates p62 ubiquitination and degradation to regulate cancer cell proliferation [129]. Moreover, Peli1 mediates p62 K63-linked ubiquitination at K7 during ischemia-reperfusion, preventing homodimerization and maintaining cardiomyocyte autophagic flux [157].

Deubiquitinating enzymes (DUBs) act as a counter-regulation of p62 ubiquitination, thereby modulating p62’s ability to engage with the autophagic machinery [131]. For instance, ubiquitin-specific protease 13 (USP13) directly binds p62 and removes ubiquitin at K7 of the PB1 domain, enhancing p62 protein stability and oligomerization, which promotes autophagy and degradation of key regulators like Keap1 [54]. Similarly, ovarian tumor deubiquitinating enzyme 7b (OTUD7B) removes ubiquitination of p62 at K7, thereby promoting p62 oligomerization and antagonizing excessive immune responses [130]. Additionally, USP14 mediates p62 deubiquitination, influencing p62 protein levels and stability, while SidE family effectors disrupt USP14–p62 interactions to inhibit xenophagy [132]. USP8 directly interacts with and removes K420 ubiquitination from p62, thereby inhibiting misfolded protein binding [131]. However, Glycyrol blocks the deubiquitinating of p62 by USP8, which promotes autophagy and maintains intestinal health [158]. Moreover, USP15 reverses RNF26-mediated ubiquitination of p62 to regulate vesicle release into the cell’s periphery [125]. Collectively, this ubiquitin–deubiquitinase axis precisely controls p62’s functions in proteostasis, signaling, and stress adaptation.

### 4.4. Acetylation of p62

Acetylation is a critical PTM regulating diverse cellular processes, including autophagy [159]. Acetylation serves as a vital regulatory mechanism in autophagy, influencing its initiation, the formation of autophagosomes, and the transcriptional regulation of ATGs [160]. p62 acetylation is dynamically regulated by various enzymes, which play an important role in biological functions. During nutrient deprivation, KAT5/TIP60 acetylates p62 at lysine residues K420 and K435 [135]. This modification disrupts the dimerization of the UBA domain, thereby enhancing p62’s affinity for ubiquitinated substrates and facilitating autophagic clearance [135]. Conversely, HDAC6 deacetylates these residues, dynamically regulating this process [135]. Beyond autophagy, p62 acetylation influences oncogenesis. Sirt1-mediated deacetylation of p62 at K295 inhibits p62 ubiquitination and degradation, thereby promoting the growth of HCCs [134]. Conversely, acetyltransferase GCN5 inhibits hepatocellular carcinoma by facilitating p62 deacetylation and subsequent proteasomal degradation by Keap1 [134]. Furthermore, p62 acetylation also contributes to DNA damage repair. p62 accumulates in the nucleus under oxidative stress and is acetylated by acetyltransferase hMOF and deacetylated by SIRT7 at the K264 site [133]. Acetylated p62 is recruited to chromatin, where it directly interacts with Apurinic/Apyrimidinic Endonuclease 1 (APE1) and enhances its endonuclease activity, facilitating base excision repair (BER) and promoting cell survival [133]. Thus, site-specific acetylation of p62 critically regulates its functions in autophagy, tumorigenesis, and genome maintenance.

### 4.5. S-Acylation of p62

S-acylation (palmitoylation) is a crucial PTM involving the attachment of palmitic acid to cysteine residues in proteins, which influences their localization, stability, and function [161]. S-acylation of p62 is dynamically regulated by zinc-finger Asp-His-His-Cys S-acyltransferase (ZDHHC19) and acyl protein thioesterase 1 (APT1) [55]. Mechanistically, ZDHHC19 catalyzes the S-acylation of p62 by mediating fatty acid attachment to the Cys289/Cys290 residues of p62, whereas APT1 reverses the acetylation of p62 [55,136]. Functionally, S-acylation of p62 enhances the localization of p62 liquid droplets on phagophore membranes, enhancing the degradation rate of p62 and the autophagy clearance efficiency mediated by p62 [55]. Furthermore, dysregulation of p62 S-acylation impairs selective autophagy, leading to obstacles in the clearance of toxic proteins and contributing to the development of Huntington [162]. Collectively, p62 S-acylation is a reversible modification that enhances the affinity of p62 for LC3 membranes and the selective autophagy flux, whereas its dysregulation is closely associated with neurodegenerative diseases.

## 5. PTMs of p62 and Oligomerization

Protein oligomerization represents a fundamental mechanism for functional diversification, enabling monomeric units to assemble into higher-order complexes through non-covalent interactions (hydrogen bonds, hydrophobic interfaces, and electrostatic complementarity) [163]. This process dynamically regulates protein functionality by modulating structural stability, enzymatic activity, and biomolecular interactions, which is critical for diverse biological processes including cellular signaling, protein quality control, and autophagic degradation [164]. The oligomerization of p62 (including self-polymerization and liquid–liquid phase separation) is the core structural feature of its multifunctionality [165,166]. PTMs integrate autophagy and signaling functions by directly regulating the oligomerization state of p62 [167]. The PB1 domain-driven oligomerization of p62 serves as a structural prerequisite for its functionality, facilitating multivalent interactions with ubiquitinated substrates and LC3 proteins to enhance cargo-binding capacity and autophagic flux [168]. Comparative studies reveal that oligomeric p62 exhibits 5–8-fold greater substrate sequestration efficiency than monomers, directly correlating with autophagic flux rates [85,165]. Structural analyses demonstrate that PB1-mediated oligomerization generates a charged molecular scaffold essential for phase separation and stress granule formation, as exemplified by K7/D69 salt bridge networks [89]. Additionally, p62 forms heterocomplexes with NBR1 through PB1–PB1 domain interactions, synergistically promoting autophagic clearance of misfolded proteins [43].

**Figure 6:**
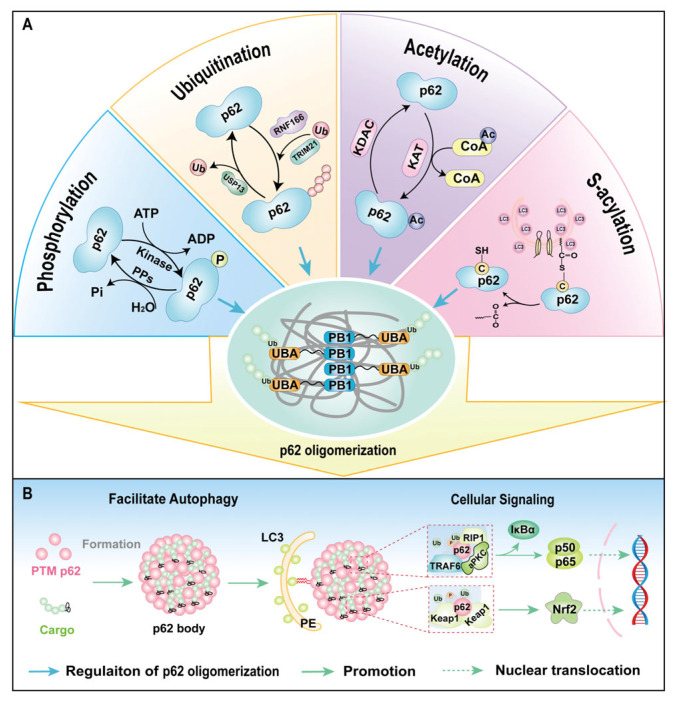
Post-translational modifications (PTMs) integrate autophagy and signaling functions by directly regulating the oligomerization state of p62. (**A**) The oligomerization of p62 is dynamically regulated by multiple PTMs, including phosphorylation, ubiquitination, acetylation, and S-acylation. (**B**) PTMs enhance the ability of p62 to bind to ubiquitinated substrates, and simultaneously integrate selective autophagy, signaling pathways, and stress responses (such as antioxidant and antibacterial responses) by altering the oligomerization state.

The oligomerization of p62 is dynamically regulated by multiple PTMs, including phosphorylation, ubiquitination, acetylation, and S-acylation (Figure 6) [169]. Phosphorylation exerts profound effects, particularly at PB1 domain residues: oxidative stress-triggered phosphorylation stabilizes electrostatic interactions between PB1 domains, enhancing self-oligomerization and facilitating the formation of p62 condensates that recruit ubiquitinated cargoes to autophagosomes [93,170]. Conversely, ubiquitination displays dual regulatory roles in p62 oligomerization: site-specific ubiquitination promotes PB1-dependent oligomerization by creating recognition platforms for ubiquitin-binding proteins [120], while K63-linked polyubiquitination at K7 by TRIM21 sterically hinders PB1 domain interactions and suppresses oligomerization [118]. This TRIM21-mediated modification sterically hinders PB1 domain interactions and redirects p62 towards proteasomal degradation pathways [118]. This ubiquitination equilibrium is dynamically modulated by USP13 and OTUD7B, which remove inhibitory K63-linked chains to restore oligomerization competence [54,130]. Acetylation critically regulates ubiquitin-binding capacity: nutrient stress-induced acetylation at residues such as K420 and K435 within the UBA domain disrupts autoinhibitory dimerization, exposing ubiquitin-binding surfaces and enhancing p62’s ability to cluster polyubiquitinated substrates while promoting integration into autophagic membranes through PB1 domain oligomerization [135]. S-acylation further refines regulation by modifying membrane affinity, enhancing p62 partitioning into LC3-positive membranes via electrostatic and hydrophobic interactions, thereby coupling oligomerization with autophagosome recruitment during proteotoxic stress [55]. These findings collectively demonstrate that p62 oligomerization is dynamically tuned through PTM-mediated structural changes, determining its dual roles in autophagy regulation and stress adaptation.

## 6. Discussion

p62 is a multidomain adaptor protein central to selective autophagy, where it acts as a bridge between ubiquitinated cargo and the autophagic machinery [83,171]. It transports these substances to autophagosomes for degradation, thereby maintaining the intracellular homeostasis [171]. This process is particularly important during nutrient deprivation or oxidative stress. p62 can act as a signaling hub to coordinate various cellular pathways, such as mTORC1 and Keap1-Nrf2 [46,150]. Furthermore, p62 enhances autophagy efficiency by forming polymers to mediate the autophagic clearance of ubiquitinated proteins. The multi-faceted regulation of p62 function by PTMs is a key determinant of the dynamic balance of autophagy. PTMs such as phosphorylation, acetylation, ubiquitination, and S-acetylation precisely control the phase separation, cargo recruitment, and autophagic degradation efficiency of p62 by altering its conformation, stability, and interaction ability. The cross-talk among phosphorylation, acetylation, and ubiquitination can influence the biological functions of p62 at multiple levels, enabling cells to cope with complex changes. This precise regulation ensures the flexibility of autophagy in responding to stress, but it is susceptible to dysregulation.

The dysfunction of p62 and its PTMs is associated with a variety of human diseases, including neurodegenerative diseases, cancer, and metabolic diseases [108,172,173]. In neurodegenerative contexts such as Alzheimer’s disease (AD) and FTLD, elevated p62 levels in cerebrospinal fluid correlate with impaired autophagic clearance, exacerbating neuronal dysfunction [174]. Notably, p62 acetylation restores its capacity to facilitate autophagic degradation of ubiquitinated substrates by modulating protein–protein interactions [135]. In HIV-associated neurocognitive disorders (HAND), p62 phosphorylation under oxidative stress amplifies neuroinflammation through increased expression of cytokines (MCP-1, IL-6, COX-2), driving disease progression [175]. In cancer, p62 overexpression and impaired degradation promote tumorigenesis, chemoresistance, and metabolic reprogramming [176,177]. PTM dysregulation disrupts p62’s role in key pathways, including the Keap1–Nrf2 axis, potentially contributing to tumorigenesis and therapeutic resistance [178,179]. For instance, dysregulation of p62 phosphorylation could continuously activate Nrf2, thereby reprogramming glucose and glutamine metabolism and promoting the survival of HCC cancer cells [173]. Oxidative damage further induces nuclear accumulation of acetylated p62, which binds APE1 to potentiate base excision repair (BER) [133]. This mechanism promotes tumor cell survival in HCCs and correlates with poor prognosis. Additionally, p62 upregulation confers resistance to therapies like 5-fluorouracil in breast cancer by activating proliferation signals and metabolic reprogramming [176]. Collectively, p62 and its PTMs represent pivotal molecular nodes linking autophagy dysregulation, metabolic reprogramming, and therapeutic resistance in disease pathogenesis.

Emerging therapeutic strategies targeting p62 or its PTMs include pharmacological inhibitors and diagnostic applications that hold promise for clinical interventions in neurodegenerative diseases and cancers. In neurodegenerative diseases, activating the autophagy pathway to promote non-canonical autophagic degradation of p62 aggregates could potentially improve neuronal function and delay disease progression [180]. Suppression of p62 expression or inhibition of its interaction with the NLRP3 inflammasome can attenuate microglial pyroptosis and neuroinflammation [175]. Additionally, altering the phosphorylation of p62 enhances the p62-Keap1-Nrf2 positive feedback loop, activating antioxidant defenses to mitigate oxidative damage in neurodegenerative diseases [95]. In cancer, targeting p62 offers the potential to overcome chemoresistance by modulating autophagy pathways and cellular signaling. Moreover, downregulation of p62 reduces IL-8 levels and increases CRC cell sensitivity to 5-fluorouracil (5-FU) and oxaliplatin (OxaPt), indicating its prognostic value as a predictor of treatment response [176]. Novel agent PTX80 targets p62 and has demonstrated the ability to reverse chemoresistance and reduce tumor growth in various malignant tumors [181]. Furthermore, targeting p62-related signaling hubs, such as the mTOR pathway, can reverse drug resistance caused by autophagy dysregulation in tumors [182]. Regarding PTM targeting, modulating PTMs represents a viable therapeutic strategy to restore selective autophagy and mitigate diseases associated with p62 dysfunction, as PTMs play critical roles in disease pathogenesis. Furthermore, numerous bioinformatics analyses and clinical tissue samples showed that p62 is abnormally upregulated in various tumors and neurodegenerative diseases, which promotes their development. The abnormal expression of p62 across tumors and neurodegenerative diseases supports its potential as a valuable biomarker [174,183]. Clinical analyses indicate that p62 levels correlate with disease progression and chemotherapeutic outcomes, such as in esophageal adenocarcinoma, where it informs treatment [184]. In neurodegenerative diseases, elevated p62 concentrations in cerebrospinal fluid serve as a non-invasive biomarker for early detection, enhancing disease control through timely intervention [174]. Collectively, p62 and its regulatory mechanisms present opportunities as therapeutic targets and diagnostic indicators, with expression levels guiding personalized strategies for cancers and neurodegenerative disorders.

In conclusion, the versatility of p62 is precisely regulated by PTMs (such as phosphorylation, ubiquitination, and S-acetylation) and oligomerization (self-aggregation, phase separation). PTMs enhance the ability of p62 to bind to ubiquitinated substrates, and simultaneously integrate selective autophagy, signaling pathways, and stress responses (such as antioxidant and antibacterial responses) by altering the oligomerization state. This mechanism plays a central role in maintaining protein homeostasis, but when dysregulated (such as p62 accumulation or mutations), it leads to the pathological processes of neurodegenerative diseases and cancer. Collectively, the dynamic regulation of PTMs underscores the central role of p62 in disease pathology and highlights its potential as a target for therapeutic interventions in autophagy-related diseases.

## Figures and Tables

**Table 1:** Selective autophagy receptors.

Selective Autophagy	Substance	Mammalian Autophagy Receptors	References
Aggrephagy	Protein aggregate	p62, NBR1, OPTN	[9,10,11]
Mitophagy	Mitochondria	p62, NDP52, OPTN, NIX, TAX1BP1, NBR1, AMBRA1, BNIP3, FUNDC1, Bcl2L13, FKBP8, PHB2, NLRX1, cardiolipin, ceramide	[12,13,14,15,16,17,18,19]
Lysophagy	Lysosome	p62, TRIM16	[20,21]
Pexophagy	Peroxisome	p62, NBR1	[22,23]
Xenophagy	Bacteria, Viral	p62, NDP52, OPTN, TAX1BP1, TRIM5α	[24,25,26,27,28,29]
Midbody autophagy	Midbody rings	p62, NBR1, TRIM17	[30,31,32]
ER-phagy	ER	FAM134B, RTN3, CCPG1, ATL3, TEX264, SEC62	[33,34,35,36,37,38]
Ferritinophagy	Ferritin	NCO4A	[39,40]
Glycophagy	Glycogen	Stbd1	[41]
Nuclear lamina autophagy	Nuclear lamina	Lamin B1	[42]
Ribophagy	Ribosomes	NUFIP1	[38]

**Table 2:** The confirmed PTMs in p62: sites of PTMs of p62 and regulators of regulation.

Site	Location	Type of PTM	Regulators
Ser28	PB1	Phosphorylation	GSK3β [92], KHK-A [93]
Ser293, Ser294	Linker	Phosphorylation	AMPK [94]
Ser349	KIR	Phosphorylation	ULK1 [95], TBK1 [96], PKA [97], mTORC1 [98], PKCδ [99], PERK [100], LRRK2 [101]
Ser403	UBA	Phosphorylation	TBK1 [102], ULK1 [103], TAK1 [104], PTK2 [105], LRRK2 [101], CK2 [106]
Ser407	UBA	Phosphorylation	ULK1 [107], TBK1 [108]
Thr138	ZZ	Phosphorylation	LRRK2 [109]
Ser207, Thr269	TB, Linker	Phosphorylation	DYRK3 [110]
Thr269, Ser272	Linker	Phosphorylation	P38δ [111], CDK1 [112], VAVC [113], CDKL5 [114]
Thr269, Ser272	Linker	Dephosphorylation	MTMR7 [115]
Ser403	UBA	Dephosphorylation	SSH1 [116]
K7	PB1	Ubiquitination	NEDD4 [117], TRIM21 [118]
K13	PB1	Ubiquitination	Parkin [119]
K91, K189	PB1, Linker	Ubiquitination	RNF166 [120]
K281	Linker	Ubiquitination	SCF^cyclinF^ [121]
K420	UBA	Ubiquitination	Keap1 [122]
-	-	Ubiquitination	TRIM25 [123], SKP2 [124], RNF26 [125], TRIM13 [126], STUB1 [127], XIAP [128], Cul5-ASB6 [129]
K7	PB1	Deubiquitination	USP13 [54], OTUD7B [130]
K420	UBA	Deubiquitination	USP8 [131]
-	-	Deubiquitination	USP14 [132], USP15 [125]
K264	NLS2	Acetylation	hMOF [133]
K295	Linker	Acetylation	GCN5 [134]
K420, K435	UBA	Acetylation	TIP60 [135]
K264	NLS2	Deacetylation	SIRT7 [133]
K295	Linker	Deacetylation	Sirt1 [134]
K420, K435	UBA	Deacetylation	HDAC6 [135]
Cys289, Cys290	Linker	S-acylation	ZDHHC19 [55,136]
Cys289, Cys290	Linker	Deacylation	APT1 [55,136]

## Data Availability

Not applicable.
